# Selecting for heat tolerance

**DOI:** 10.1093/af/vfy033

**Published:** 2019-01-03

**Authors:** María J Carabaño, Manuel Ramón, Alberto Menéndez-Buxadera, Antonio Molina, Clara Díaz

**Affiliations:** 1 Departamento de Mejora Genética Animal, INIA, Madrid, Spain; 2 Centro Regional de Selección y Reproducción Animal (CERSYRA)—Instituto Regional de Investigación y Desarrollo Agroalimentario y Forestal de Castilla-La Mancha (IRIAF-JCCM), Valdepeñas, Spain; 3 Departamentos de Genética y de Producción Animal, Universidad de Córdoba, Córdoba, Spain

**Keywords:** heat tolerance, breeding tools

**Implications**

- Identification of heat tolerant animals is challenging due to the complexity of heat stress response and the antagonism between heat tolerance and productivity. Advances are needed to: 1) find fine phenotypes to identify heat tolerant animals on farm; 2) develop methods to combine the knowledge from all “-omics” technologies.
- Breeding strategies to improve heat tolerance will depend on the production system. Systems that can provide enough resources to ensure high productivity will benefit more from including heat tolerance in the breeding programs of specialised breeds. In contrast, production systems with scarce resources will benefit more from crossing with local stock.

## How Do We Measure the Heat Tolerance of Animals?

It is not obvious how to define a heat-tolerant animal. In principle, a heat-tolerant animal is one that maintains homeothermy under high environmental heat loads. However, from a livestock breeding point of view, maintaining productive and reproductive levels under hot conditions may be the target. Maintaining homeothermy under hot conditions depends on the animal’s ability to balance thermogenesis and heat dissipation. Several measures have been proposed as criteria to identify heat tolerant animals; these include body temperature, respiration rate, heart rate, and sweating rate. Animal performance under heat stress is a way of measuring the overall ability of the animal to cope with heat. Hair and coat characteristics including hair shedding rate and body surface to mass ratio are related to the animal’s ability to dissipate internal heat. These measures have also been proposed as heat tolerant traits (Gray et al., 2011). Several biomarkers such as blood parameters (Van Goor et al., 2016) or diverse molecules associated with the heat stress response have also been proposed as indicators of heat stress in livestock (Min et al., 2017).

From the perspective of the implementation of a selection program for heat tolerance, measures that can be collected easily under farm conditions at a low cost are needed. Most of the efforts to implement genetic evaluations for heat tolerance have used performance recording under heat stress, following the original developments of Ravagnolo et al. (2000). Information of weather conditions (temperature and humidity most often combined in the temperature humidity index proposed by NRC, 1971) on the day or previous days of performance recording is merged with performance records to quantify the reaction of animals to heat loads in terms of productivity. This approach has the advantage of low cost, since performance recording is already available in livestock breeding schemes, but it also has some drawbacks. The first limitation is due to the ability to produce accurate measures of heat tolerance from existing recording schemes, which are not designed to capture the heat stress response. An example of this is shown in Freitas et al. (2006), where the heat stress response was largely underestimated when comparing the monthly recording (normally used in milk recording) to a weekly recording. Another limitation is related to the antagonism between productive level and heat tolerance. Thus, selecting animals with smaller slopes of decay in performance at high temperatures may decrease the productive level in the population, as it will be later illustrated.

Physiological traits such as body temperature or respiration rate are considered as gold standard measures for heat tolerance, but their use in large-scale selection programs is still limited because it is expensive to collect these measurements. Advances in the development of devices that can produce measures automatically at a low cost might change the possibility of using these types of measures in breeding programs in more intensive production systems (Koltes et al., 2018).

Quantification of levels of heat stress biomarkers could be achieved at a low cost in dairy populations through the use of mid-infrared spectroscopy that are routinely obtained to determine the main components of milk. The milk spectra could be calibrated to quantify the level of metabolites or other substances identified as biomarkers of heat stress, providing a potentially inexpensive tool to identify heat tolerant animals. However, the complexity of the heat stress response makes the selection of a reduced number of key biomarkers a difficult task. Recently, Hammami et al. (2015) explored the use of mid-infrared spectroscopy to assess profiles of milk fatty acids as possible biomarkers for heat stress in dairy cattle.

## The Genetic Component of Heat Tolerance

As described above, genetic selection might be a cost-effective tool to improve thermotolerance of animals. However, for genetic selection to be effective, it is necessary to have a deep knowledge about the genetic basis of the animal’s response to heat stress. Many studies have used different genetic tools to study the genetic basis of heat stress including, classic quantitative genetics as well as the more recent “omics” technologies. All of these technologies have the main goal of understanding what makes some animals more thermotolerant than others.

### Genetic variability of heat tolerance and genetic evaluations

**Figure 1.**
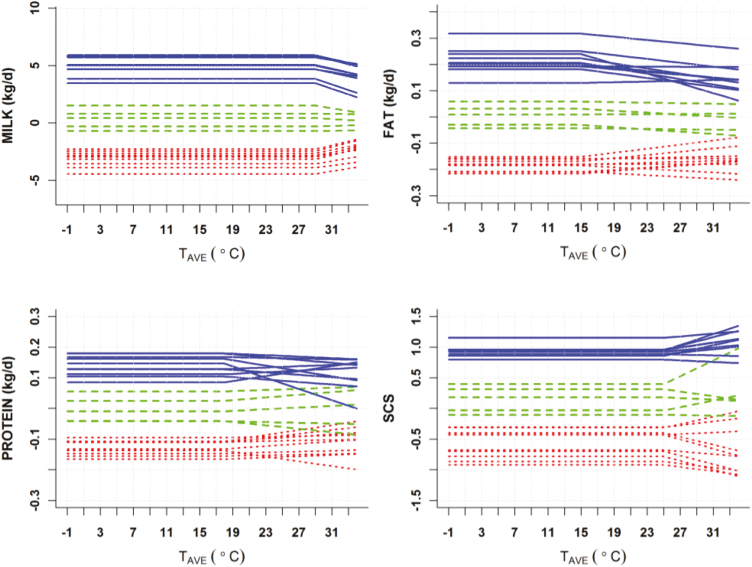
Estimated individual deviations from the average population response in productive traits, milk, fat, protein and somatic cell score (SCS), to increasing values of daily average temperature (T_AVE_) under a broken line model for top (blue), average (green) and bottom (red) animals according to the level of each trait (Source: Carabaño et al., 2014).

**Figure 2.**
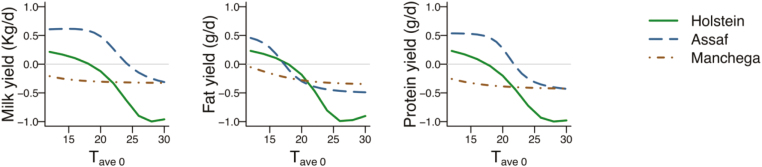
Correlations between estimated values for production level (milk, fat and protein yields) and thermo-tolerance (slope of production decay) along the scale of average daily temperatures (T_ave0_) in three dairy breeds: Holstein cattle, International Assaf and Local Manchega sheep.

Most of the studies designed to determine the genetic value of heat tolerance of animals have focused on modeling the genetic component of performance under high heat loads as described by Ravagnolo et al. (2000). This approach describes the genetic component of the reaction to heat stress in performance with the so-called broken line model. The broken line model is defined by two parameters: 1) the thermoneutrality threshold and 2) the slope of decay in production after passing this threshold as a consequence of heat stress (Bernabucci et al., 2014). Alternatively, Brügemann et al. (2011), Menendex-Buxadera et al. (2012), and Carabaño et al. (2014) proposed the use of polynomials of second or third order to describe the norm of reaction of milk production across the heat load scale. Polynomial functions provide a more flexible approach than broken line models and allow for a smoother transit from thermotolerance to heat stress, instead of an abrupt change after the thermoneutrality threshold in broken line models. With this approach, steeper slopes at higher temperatures are accommodated, instead of a constant slope of decay in the broken line model, as might be expected to occur in reality. Reaction norm models using performance (both productive or reproductive) records and meteorological information have been extensively applied to measure heat tolerance in dairy or meat oriented production (Menéndez-Buxadera et al., 2012; Biffani et al., 2016; Bradford et al., 2016). One of the main issues in the application of this approach is how to combine climate variables in the models to define the amount of heat load that is received by the animals. A number of studies have dealt with the use of alternative definitions of indices that combine temperature, humidity and additional meteorological variables such as wind speed or insulation (Gaughan et al., 2012). The definition of the lag between the date of recording the animal’s performance and the date for which weather conditions better determine the subsequent animal’s response in performance has the same importance as the weather variables to be included in a heat load index (Bernabucci et al., 2014, Carabaño et al., 2014, Ramón et al., 2016). Another important issue is to determine the selection criteria derived for each model. In the broken line model, both the thermotolerance threshold and the slope of response of each individual could be used as selection criteria. However, the estimation of individual thresholds has been found to be troublesome from a computational point of view (Sánchez et al., 2009). Most applications of this model assign a predetermined value for the threshold and only the slope is estimated for each animal. The large estimated genetic correlation between threshold and slope [−0.95 in Sánchez et al. (2009)] indicates that selecting animals with less negative slope of response under heat stress will also result in higher thermotolerance thresholds. When higher than first-order polynomials or other functions are used to describe the norm of reaction to heat stress, the definition of selection criteria is less obvious. Alternative selection criteria might be the slope of the individual polynomial curves under moderate or severe heat stress or principal component values derived from the eigen decomposition of the covariance matrix of the random regression coefficients for the genetic component (Carabaño et al., 2014; Macciotta et al., 2017). All mentioned studies dealing with estimation of the genetic component of productive response under heat stress have shown variability across animals, indicating that genetic selection is possible. Figure 1 shows the estimated genetic deviation from the mean response to increasing temperatures of top, average, and bottom cows sorted by the level of milk, fat, protein, and somatic cell count using a broken line model. The figure illustrates the variability in genetic response of several animals and the reranking of animals at different temperatures, which indicates a certain degree of genotype by environment interaction. It can also be observed in this figure that the top animals for the level of the trait tend to show larger decays that an average animal, while the worst animals tend to have less negative responses than the average, which represents the antagonism between productivity and heat tolerance. The degree of antagonistic relationship in different types of dairy populations is illustrated in Figure 2. This figure shows the correlation between the estimated values for level of production and the rate of production decay under heat stress in three dairy populations: Holstein dairy cattle, the international breed Assaf and the local breed Manchega of dairy sheep. For the Holstein, which has been very intensively selected for milk production, correlations between milk production potential and the rate of production decay at successively higher temperatures becomes nearly −1 under heat stress, meaning that animals with a larger potential to produce milk will be the ones showing more negative slopes of decay. In contrast, these correlations are much lower for both sheep breeds, which implies that animals with an overall high potential for production and good heat tolerance is cumbersome. Selection indices with appropriate weighing for production and heat tolerance might be used to overcome the antagonistic relationship between those two traits. However, determining the appropriate economic weight for heat tolerance may be complex because of the difficulty of identifying all the animal performance parameters that are altered by heat stress and quantifying the associated economic loss.

Determination of the genetic component for other measures of heat tolerance has been mainly focused on body temperature and respiration rate (Dikmen et al., 2012; Gourdine et al., 2017; Van Goor et al., 2016). The heritability estimates ranged from 0.10 for cloacal temperature in chicken and 0.17 in the dairy cattle study for rectal temperature to values more than 0.30 for both rectal or skin temperatures and respiration rate in lactating sows. Genetic variability has also been detected for this type of measure of heat tolerance, making selection theoretically feasible but impractical because of the high cost of measuring these parameters.

Overall, up to now, the attempts to produce genetic evaluations to select heat-tolerant animals have been based on analyses of performance under heat stress. Examples of these attempts can be found for dairy (Bohmanova et al., 2005) and beef cattle (Bradford et al., 2016). More recently, a genomically enhanced evaluation has been developed for dairy cattle in Australia (Nguyen et al., 2016).

### Omics to understand the genetic component of heat tolerance

**Figure 3.**
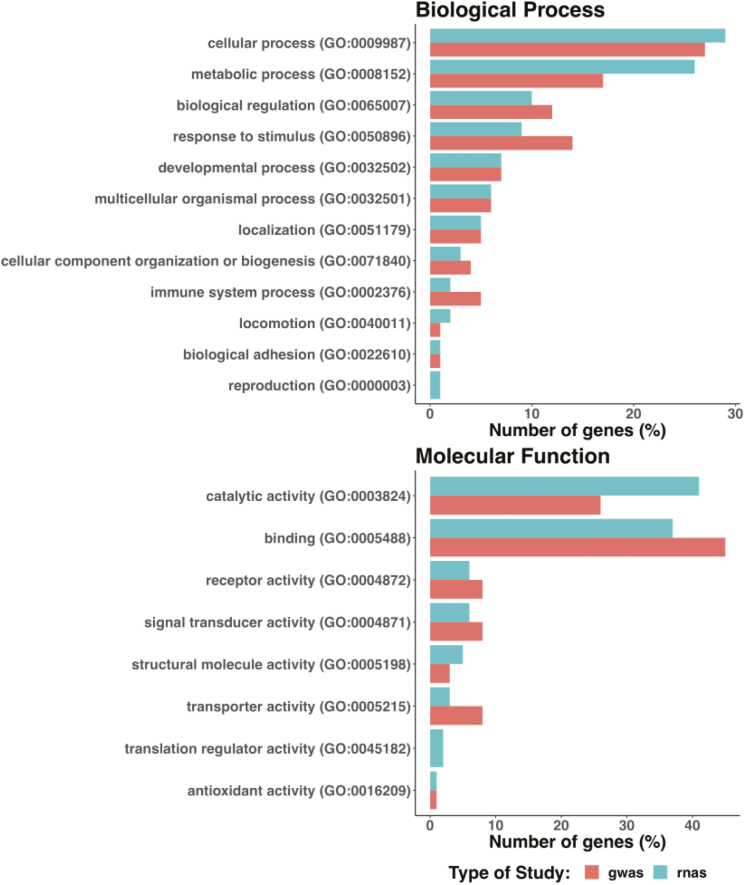
Gene ontology (GO) terms of genes reported in the literature of genome wide association (gwas) and transcriptomic (rnas) studies to be involved in the response of animals to heat stress. Bars show the number of genes (percent of total) for biological processes or molecular functions obtained from the GO analysis using Panther (http://pantherdb.org/).

Quantitative genetic studies suggest a non-negligible genetic component of thermotolerance, which somehow is reinforced by a number of studies including “omic” information to gain knowledge about the genetic mechanisms behind the animal’s response to heat. Three main types of studies can be found in the literature: 1) association studies of polymorphisms at specific genes and genome-wide association analysis (Macciotta et al., 2017); 2) genome comparison between adapted and nonadapted breeds/species to harsh environments (Chan et al., 2010) and 3) differential expression analyses (Chauhan et al. 2014). A literature review of these studies has provided over 431 candidate genes for the heat stress response. Results from a functional analysis of those genes using Panther v.11 (Mi et al., 2017) is shown in Figure 3. In general, genes reported for all three types of studies are functionally classified into similar gene ontology terms which is a form of validating that the association analysis are pointing to the correct genomic regions (i.e., the ones that show differential expression under heat stress vs. thermoneutrality). Moreover, the pseudo-phenotypes used to measure heat tolerance and defined in different species for association studies are good proxies and are able to capture the sensitivity of animals to heat loads.

In Figure 3, biological processes are described by their outcome or ending states that are normally achieved by a set of molecular functions carried out by specific gene products. As part of the biological processes, those related with response to stress, as well as metabolic processes, biological regulation, or immune responses are the most represented. The heat stress response has been previously shown to result in increased catabolism, oxidative stress, and jeopardized immune response (Bernabucci et al., 2010), which agrees with the proposed candidate genes and their ontology.

Apart from the functional analysis of candidate genes for regulation of the heat stress response, we want to highlight families of genes that are present in association and differential expression studies. The most represented families are the heat shock proteins and DnaJs. DnaJs proteins seem to be crucial partners of the heat shock protein-70 (Qiu et al., 2006) and they are important for protein translation, folding, unfolding, translocation, and degradation. In addition, genes from interleukin, chemokine, and fibroblast growth factor families are found. These families are mostly involved in immunological and inflammatory processes, which are one of the major consequences for animals exposed to harsh environments (Bernabucci et al., 2010). Interestingly, heat shock factor-1 has also been found in several studies. Heat shock factor-1 is an evolutionarily conserved transcription factor that binds to the promoter regions of heat shock proteins to regulate their stress inducible synthesis in response to the environment. In summary, reports in the literature describe the complexity of the effects of heat stress on the physiology of a production animal and, therefore, illustrate the difficulties of using genomic information to select thermotolerant animals.

Apart from the numerous candidate genes that have been associated with regulation of the heat stress response, the slick hair gene deserves special attention. The slick hair gene, located on chromosome BTA20, is responsible for a smooth and short hair coat, confers thermotolerance to the animal, and is associated with an improved capacity for heat dissipation. Introgression of the slick hair gene (present in Senepol cattle and some lines of highly productive Holstein cattle) has been shown to produce animals with lower body temperatures and smaller declines in production under hot conditions (Dikmen et al., 2014; Ortiz-Colón et al., 2018). Slick positive Holstein bulls are already marketed by the artificial insemination companies. However, slick hair may decrease the ability of animals to cope with cold temperatures, which may be important in climates that include hot and cold periods.

## Breeding Strategies

Breeds that originated in warm climates show adaptive advantages to heat stress compared with breeds that originated in temperate areas. Many studies have shown that under heat stress, breeds from warm climates have lower respiration rates, body temperature, or sweating rates and better reproductive performance than breeds from temperate climates (Hansen, 2004; Berman, 2011; Gourdine et al., 2017). Another general characteristic of locally adapted breeds is the low level of production. Berman (2011) and Hoffman (2010) reviewed the advantages and disadvantages of using breeds locally adapted to extreme conditions to improve tolerance to heat stress. One of the conclusions of Berman (2011) is that low productivity of adapted breeds might be a constitutional characteristic of these breeds since several studies show that breeds from warm climates and their crosses with selected breeds tend to favor fat deposition and body condition score over milk production when improved feeding is provided. The fact that fat deposition might be an advantageous constitutive characteristic associated with large seasonal variations in grazing conditions normally present in warm climates could be the evolutionary reason for this adaptation strategy. If this were the case, improving productivity in breeds adapted to harsh conditions might be impaired by this characteristic, and, on the other hand, the use of these breeds to improve heat tolerance of selected breeds might confer an undesirable genetic background in addition to the desired heat tolerance. Moreover, the enormous gap in productivity between selected and locally adapted breeds questions the profit from using these breeds to improve thermotolerance of more productive breeding stock when farm resources and animal health are not limiting the survival of highly selected breeds.

**Figure 4.**
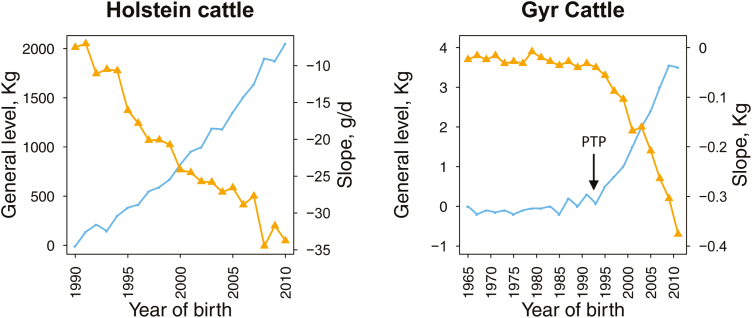
Estimated genetic trends in two dairy cattle breeds: Holstein (*Bos Taurus*) amd Gyr (*Bos indicus*). Lines show genetic trends for milk production (blue) and heat tolerance (orange). For Gyr cattle, year of first result of progeny test program (PTP) is marked by and arrow. Source: Carabaño et al., 2017 (left) and Santana et al., 2015 (right).

Overall, there are two main scenarios. When the production system is sufficient to provide adequate feeding, management, heat mitigation, and controlled parasite and pathogenic environment, selection for heat tolerance within highly productive breeds is likely to offer far more opportunity than improving local breeds. On the other hand, crossing of local and selected breeds and selection for productivity and monitoring of heat tolerance seems to be the best option to improve productivity in production systems that cannot provide mitigation for heat, adequate nutritional conditions or control of parasites and other pathogens. Figure 4 illustrates the results of current selection programs on milk production and heat tolerance (slope of production decay) in two populations of dairy cattle: 1) Holsteins raised in Mediterranean conditions (Carabaño et al., 2017) and 2) Gyr in the tropics (Santana et al., 2015). For both populations, genetic selection to increase milk production has had an associated negative response in the animal’s ability to cope with heat stress. Similar results have been shown in Carabaño et al. (2017) for local goat and sheep breeds in Spain. Thus, even for locally adapted breeds, heat tolerance has to be monitored when selection for productivity is implemented in production systems affected by heat stress.

## Conclusions

Heat stress is a complex phenomenon that triggers a number of response mechanisms in animals that have a negative effect on farm profitability. Of all the actions that farmers can implement to adapt to the challenge of heat stress, genetic selection can provide a cost-effective and efficient tool to improve the resilience of farms to hot conditions. Up to now, selection procedures were based on estimating the decrease in production under heat stress by using information from current farm recording schemes and meteorological information on the day of recording. Substantial genetic variability has been observed in an individual animal’s response to increased heat loads, with a moderate degree of genotype by environment interaction, which implies that animals that are the best producers under comfort may not be the best animals under heat stress. However, this approach has two major drawbacks: 1) inaccuracy of the individual estimate of the animal’s ability to maintain its level of productivity under heat stress because of the scarcity of individual records along the heat load scale and 2) antagonism between the productive and heat tolerance criteria. Thus, it is necessary to improve heat tolerance phenotyping to produce more accurate measures to identify heat tolerant animals and increase our understanding of the underlying genetic mechanisms of heat tolerance that can be used in selection programs.

A large amount of knowledge is being accumulated about the underlying mechanisms of the heat stress response from “omics” studies. Many candidate genes and potential biomarkers have been proposed from DNA, RNA, and metabolomics studies, but there is still work to be done to combine this accumulated knowledge to provide selection tools to improve heat tolerance in breeding schemes.

Optimal breeding strategies to improve heat tolerance of livestock (i.e., selecting for heat tolerance within highly productive populations vs use crossbreeding or introgression involving local and selected breeds) will depend on the farm resources (including nutrition, management, and investment capacity) and level of parasite or other pathogen challenges of the production system.

## About the Authors

**María J. Carabaño**, PhD, Animal Breeding (Cornell University), is a senior researcher in the National Institute for Agriculture Research and Innovation of Spain. She has a background in genetics and animal breeding research and transfers research results for the improvement of genetic evaluation systems. Recent research interests have focused on the genetic background of heat tolerance in dairy, beef, and other ruminants. Corresponding author: mjc@inia.es

**Manuel Ramon** is a senior researcher at the Regional Center of Reproduction and Animal Breeding (CERSYRA–IRIAF) in Valdepeñas, Spain. He is primarily interested in the genetic background of the main productive and reproductive traits of interest in ruminants. He also enjoys modeling and bioinformatics applied to genetics.

**Alberto Menéndez-Buxadera** is a visiting professor at the University of Córdoba, Spain. He has taught Animal Breeding courses and has participated with several research groups from Latin American and European universities. His current research interests are genotype by environment interaction, use of longitudinal models for genetic evaluation, and selection of animals more adapted to very heterogeneous environmental conditions.

**Antonio Molina** is professor of Genetics at the Veterinary Faculty of Cordoba, Spain. His research topics include: 1) development of animal breeding programs and their implementation in local breeds of domestic animals, 2) management of genetic variability in conservation programs, 3) genetic markers for breed genetic identification and traceability, 4) detection and gene identification for traits of economic interest in local breeds of domestic animals, and 5) quantitative genetic analysis of the effects of heat stress. Recent research has focused on the genomic evaluation of domestic animals.

**Clara Díaz**, PhD in Animal Science from Virginia Tech, is a senior researcher in the Animal Breeding Department at The National Institute for Agriculture Research and Innovation of Spain. Her background is in Quantitative Genetics and Animal Breeding. Currently, her major interest is in the use of “omics” for the characterization of local beef cattle breeds and the dissection of the complexity of animals’ response to stress as a tool to contribute to animal welfare and sustainability of production systems.
